# The Impact of Hispanic and White Group Cues on Attitudes Towards the Violation of Generic Norms

**DOI:** 10.1371/journal.pone.0083154

**Published:** 2013-12-27

**Authors:** Mirjam Cranmer, Skyler J. Cranmer

**Affiliations:** 1 Social Science Research Institute, Duke University, Durham, North Carolina, United States of America; 2 Department of Political Science, University of North Carolina at Chapel Hill, Chapel Hill, North Carolina, United States of America; 3 Alexander von Humboldt Fellow, University of Konstanz, Germany; Mälardalen University, Sweden

## Abstract

While much work in political science has examined the impact of racial cues on individual perceptions, we know little about how individuals evaluate members of minority outgroups on issues that are not linked to stereotypes. We measure the impacts of Hispanic and White cues on individual assessments related to a stereotype-independent norm violation: alcoholism. We test three competing theories – cognition, intergroup emotions, and social identity – using a population-based vignette experiment included in the General Social Survey. Our results contradict much of the literature, but keep with social identity theory's predictions. Hispanic alcoholics, when Hispanics constitute the outgroup, are assessed less negatively than White alcoholics in the ingroup, the latter experiencing what is called the black sheep effect. The black sheep effect occurs when ingroup members are more punitive towards members of the ingroup than the outgroup. However, the black sheep effect does not extend to measures that are more consistent with outgroup stereotypes, such as violence or money mismanagement; Hispanic alcoholics are evaluated more negatively than Whites on these measures. The implication is that the effect of racial cues depends strongly on issue linkages to group stereotypes.

## Introduction

A great deal of work in political science has examined the determinants of attitudes towards racial minorities and found that, under certain circumstances, racial cues can be a potent means to influence one's perceptions of groups and their members [1]–[10]. A majority of this work has focused on general perceptions of racial/ethnic groups, often using cues consistent with stereotypes of the group or focusing on issues with which it is typically associated. For example, a substantial body of work focuses on the extent to which issues, typically welfare or crime, are racialized [2], [7], [11], [12]. A result of the focus on racial groups and racial issues is that evaluations of minority groups and stereotypical minority issues are often confounded. This highlights the fact that we know little about the deeper process by which individuals evaluate members of an outgroup minority: is the evaluation based on the group cue or the issue? For instance, is a Hispanic evaluated negatively because he is Hispanic or because of an individual action he has taken?

Examining the violation of generic norms, norms that apply equally across racial groups and their members [13]–[15], at the individual level allows us to identify whether an individual is evaluated negatively because of his/her actions or because of minority cue-activation. Such an identification is difficult, if not impossible, for racialized issues. Thus, we deepen our understanding of the foundations of minority evaluations by considering how racial attitudes and cues might affect evaluations of non-racialized behaviors and beliefs.

Here, we examine the role of Hispanic and White cues on individual assessments of blame related to the generic norm violation of alcoholism. We do so using data from a vignette experiment included as part of the population-based General Social Survey. After considering how cognition based, intergroup emotion based, and social identity based theories predict different results, we find robust support for the effects predicted by social identity theory (SIT). Specifically, we find that the “black sheep” effect, where generic norm violators from the ingroup are judged more harshly than those from the outgroup, pervades. However, the black sheep effect does not extend to issues that are more consistent with outgroup stereotypes, such as violence or money mismanagement. Hispanic alcoholics are evaluated more negatively than Whites on these measures. Overall, the results suggest the effect of racial cues depends strongly on issue linkages to group stereotypes. This, in turn, implies the need for a more dynamic approach to the study of affect, group cues, and blame that accounts for specific classes of issues.

## Racial Cues, Affect, and Cognition

Three major theories – based on cognition, appraisal, and social identity – address how individuals react to social/racial outgroups and the violation of norms. Each of these theories predict substantially different patterns of response by members of the ingroup. Our empirical objective below is to identify which predicted pattern of response is best supported by the data.

The cognition inspired approach suggests that the impact of outgroup cues can be moderated by counter-stereotypical cues because evaluations are made based on meaning. Valentino, et al. [10] find that suggesting to subjects that Blacks deserve government resources dampens racial priming. According to Peffley & Hurwitz [12], “judgments are linked to the stereotypes when, and only when, the case at hand fits the image.” For example, laziness and violence are seen as central attributes of racial stereotypes, and the effects of these perceptions spill over onto related issues [16]. The extent to which welfare is racialized [2], [7], [11], [12] can be explained by the perception of Black people as being lazy, undependable, overly self-interested, and demanding of society [2]. The racialization of criminality, from the cognition perspective, follows from the stereotypical image of Blacks as being aggressive, thus suggesting they engage in more violent criminal behavior than other groups [12].

Appraisal based theories, such as intergroup emotions theory (IET), attribute substantial power to outgroup cues. This suggests that certain outgroup cues produce distinct emotional responses [17]. For example, Parker Tapias et al. [18] show that the cue “Black” activates appraisal-consistent emotions (anger) that can be transferred to unrelated stimuli. Also, the cue “gay” elicits enhanced disgust. According to this view, reaction to an outgroup “should influence emotions toward any people, events, and objects encountered after the outgroup category is activated” in ways that are congruent with the reaction's appraisal theme [18]. It should be noted though, that IET is not limited in scope to the elicitation of negative emotions, but can also elicit positive emotions.

Studies based on social identity theory (SIT) propose a dynamic relationship between individual group members and the group as a whole that helps to square an apparent contradiction in the literature. While the intergroup emotions literature would predict that racial outgroups trigger qualitatively distinct, but homogenous and negative, evaluations of outgroup members that violate a generic norm because of the tendency for congruent appraisal [13], SIT posits that the violation of a generic norm can lead to more favorable evaluations of outgroup members relative to ingroup members. SIT suggests that differentiation among individuals within groups is motivated by ingroup identification and the desire for positive ingroup distinctiveness [19].

One implication of SIT is that ingroup and outgroup deviants are judged differently, even when they are deviating on the same generic norm. If a generic norm is violated by an ingroup member, that member is evaluated more negatively than an outgroup member would be for the same deviation. This extra-punitive chastisement of ingroup members is known as the black sheep effect [13]–[15]. The black sheep effect suggests that the motivation for derogation is part of an inclusive reaction and particularly prominent if ingroup closeness is high [20]. Ingroup deviants are perceived more negatively because they pose a threat to the ingroup's positive image of itself. The black sheep effect tends to prevail at the individual level despite the negative evaluation of the outgroup as a whole. Therefore, the evaluation of individual action is biased by group membership, but in a different direction than the intergroup emotions theory would predict.

Each of these theories suggest different hypotheses with respect to blame when applied to the case of generic norm violation in the context of a racial ingroup/outgroup cue. The cognition based approach predicts that the norm violation will not influence blame attribution differently across racial groups because generic norms are not racialized and the norm violation itself is a more immediate cue for blame assessment than race [10]. Intergroup emotions theory predicts that outgroup members will be blamed more than ingroup members if a generic norm is violated because of the (affective) outgroup cues that are activated whenever any norm, be it generic or not, is violated by a member of the outgroup [17]. Lastly, SIT predicts a black sheep effect in such situations: ingroup members are blamed more than outgroup members because the norm violation reflects poorly on the group to which the evaluator belongs [13]. Below, we examine which of these theories is supported in a situation where White respondents make blame attributions for the generic norm violation of alcoholism in the presence of White and Hispanic racial cues.

## Materials and Methods

We make use of a population-based vignette experiment originally developed for studying the American public's knowledge of and response to Mental Health issues [21], [22]. This series of vignette experiments was included in the General Social Survey (GSS), a representative survey of the U.S., in 1996 and 2006. The GSS is a public use data set, hence no IRB approval was required for this study. The survey is conducted by the National Opinion Research Center (NORC) at the University of Chicago and is IRB approved at that stage. Subjects participating in the GSS give consent that their anonymized responses become part of the public use data set. The vignette experiments cover 90 unique conditions by manipulating race, gender, education, and five mental health issues: alcohol dependency, major depression, schizophrenia, drug addiction (1996 only), and the control issue of no-problem.

Alcohol dependency deviates from the other health conditions in the sense that it is an issue where blame attributions are made more readily. The vignette describes an individual and their problem, without explicitly labeling the person an alcoholic, attributing the problem to him/her or attributing the problem to the individual's circumstances. The no-problem conditions served as control conditions for each race (the exact vignette wordings can be found in the Appendix).

We restrict the sample of subjects to Whites, the best represented racial group in the survey, in order to make racial in- and outgroup effects more clear. These subjects include individuals exposed to the 12 alcoholism and no-problem conditions for White and Hispanic males, a total sample of 658. The average subject was 45.7 years old with 13.7 years of education and a $20,000–24,999 household income. Female subjects constitute 53.7 percent of the sample, 42.1 percent self-identified as Republicans, and 36.1 percent as Democrats. These proportions are comparable to the Census Bureau data [22].

We use Hispanics as the racial outgroup, rather than Blacks, which is used more often in the literature, because the question wording for Blacks in the GSS is inappropriate for the purposes of this study. The post-treatment questions included a visible group tag only for Hispanics (Juan), and not Blacks (John). Therefore, when asked about the Black-vignettes, the respondent does not receive a racial cue. Conversely, questions about the Hispanic vignettes include the obvious racial cue of “Juan.” The “outgroup tag” is important to test intergroup emotion theory, which requires outgroup activation [18]. A test of the GSS data on the Black-vignettes produced, unsurprisingly, less pronounced effects.

The GSS design we use offers several advantages. First, alcoholism is not strongly related to race or a race-related issue, such as immigration. One might reasonably expect alcohol dependence to elicit blame across races, thus making it a generic norm matching the aim of this study. This claim is supported by Kantor [23] who finds that “[h]eavy drinking per se is associated similarly in Hispanic-American and Anglo-American families” (p. 57). Because a condemnation of alcoholism is an ingroup norm for both Hispanics and Whites, it is a generic norm between them. Furthermore, the construction of the alcoholism-related questions allows us to trace prejudices in a subtle manner similar to implicit priming [6]: race is explicitly mentioned in the treatment vignette but the only racial cue in the post-treatment questions is the hypothetical individual's name, the questions focus explicitly on alcoholism. Lastly, the population-based vignette experiment combines the external validity of large-scale representative surveys and the internal validity of experiments, making it ideal for drawing inferences about causal processes in populations [24].

The experimental design allows us to directly assess the effect of introducing a racial cue on blame attributions associated with the violation of a generic norm, as well as the more simple effect of a racial cue in a no-problem condition. By comparing the racial ingroup, White, norm-violating condition (condition 2 in the Appendix) to the racial outgroup, Hispanic, norm-violating condition (condition 4), we can estimate the impact of norm-violation across groups. The design also allows us to control for the possibility of sole cue priming: were we to simply compare ingroup norm violating and outgroup norm violating, we could not rule out the possibility that it is the mentioning of Hispanics or Whites, rather than the racial cue connected to the generic norm-violation, that drives the results. By comparing the no-problem conditions (condition 1 and 3), we can test directly for the importance of racial cues. Lastly, the design can assess the relative importance of blame and race. While it has been found that perceived intention is a decisive predictor of opinions [25], we can assess the relative contribution of racial cue exposure and perceived intention to violate norms.

The questions, presented to the subjects after exposure to treatment/control, include measures of blame attribution, social distance, and stereotypical assessments. The attribution of blame for the norm violation is measured by asking respondents how likely it is that the person described in the vignette's situation is caused by his “own bad character.” Blame attribution is measured on a 4-point scale ranging from 1 (“not at all likely”) to 4 (“very likely”). Social distance was assessed by asking respondents how willing they would be to have the person described in the vignette 1) work closely with them on a job; 2) live next door; 3) spend an evening socializing; 4) marry into the family; and 5) as a friend. This willingness was measured on a 4-point scale ranging from 1 (“definitely willing”) to 4 (“definitely unwilling”). Stereotypical assessments were measured in two ways. First, by the perception of dangerousness toward others: this measure asked respondents how likely is it that the person in the vignette would “do something violent toward other people” (from 1, “not at all likely,” to 4, “very likely”). Second, by perceptions of money management ability: respondents were asked, regarding the protagonist of the vignette, “how able is [John/Juan] to make his own decisions about managing his own money” (from 1, “very able,” to 4, “not able at all”).

Respondents' age (in years), sex (coded 1 for female, 0 for male), education (coded 1 for at least a high school degree, and 0 otherwise), and income (12 income categories) were included as controls. Because the GSS adopted a sub-sampling design to capture non-respondents after 2004, we follow Pescosolido et al. [22] and include a weight for the selection of one adult per household. However, the results are similar if the weighting variable is omitted.

## Results

### Manipulation Check

In order to empirically test whether alcoholism represents the violation of a generic norm (“not to be an alcohol addict”), we assess the respondents' evaluations of the seriousness of the vignette protagonist's problem. The results confirm that the seriousness of the alcohol problem is seen as “very serious” for both the White and Hispanic protagonist, thus representing a norm violation that applies across groups. On a scale from 1 to 4, “very serious” to “not at all serious,” vignettes with a White protagonist receive a mean (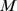) score of 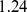 (SD 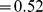) and those with a Hispanic protagonist receive a mean score of 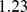 (SD 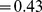) (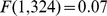; 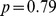). The no-problem condition was, on average, considered “not very serious” for Hispanic (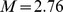 (SD 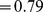)) and White (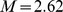 (SD 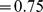)) protagonists alike, but leaning towards “somewhat serious” (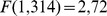; 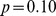).

### The Black Sheep Effect

We find that, although individuals violating norms are generally blamed more than individuals not violating a norm, White subjects blame a White alcoholic for his condition more than they do a Hispanic alcoholic (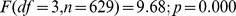). Responding to the question of how likely the protagonist's condition is caused by his own “bad character” using a scale ranging from “not at all likely” (1) “very likely” (4), White subjects produce a mean of 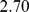 (SD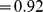) for a vignette presenting a White alcoholic and a mean of 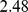 (SD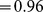) for a vignette featuring a Hispanic alcoholic (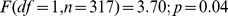). The no-problem vignettes produce lower scores across race categories, with subjects leaning towards the assessment of “not at all likely,” but there is not a statistically significant difference in blame by race 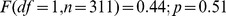). Table 1 presents the full summary statistics.

**Table 1:** Means and Standard Deviations Showing the Black Sheep Effect.

	Mean	Std. Deviation	*N*
White No-problem	2.27	0.96	158
Hispanic No-problem	2.20	0.88	154
White Alcoholic	2.70	0.92	163
Hispanic Alcoholic	2.48	0.96	155

Measures are taken on a 4-point scale in response to the question of how likely it is that the vignette protagonist's condition is caused by his “bad character.” 1 =  “not likely at all” and 4 =  “very likely”.

The social distance measures also provide some support for the black sheep effect. Table 2 presents summary statistics related to the social distance measures by treatment category. Subjects were less willing to make friends with 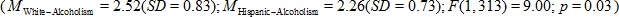 and work closely on a job with 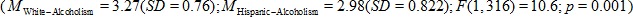 a White alcoholic than a Hispanic alcoholic. Living next door to the protagonist, spending time socializing with him, and having him marry into the family, however, do not show discernible differences across race, but do show less willingness to engage with alcoholics. Further we created a single social distance scale by taking the mean across all five social distance measures. We find that subjects were somewhat more willing to engage socially with White alcoholics 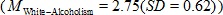 than Hispanic alcoholics 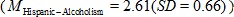. The difference between these tendencies is notable, though its 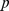-value falls between the two traditional thresholds of 0.05 and 0.10 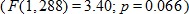. Generally, racial differences in the no-problem conditions are statistically indistinguishable from zero, while all of the measures that imply a direct relationship with the protagonist show statistically significant differences in attitudes towards the White and Hispanic alcoholics.

**Table 2:** Summary Statistics for Social Distance.

		Mean	Std. Deviation	*N*
Socializing	White No-problem	1.88	0.66	155
	Hispanic No-problem	1.86	0.79	152
	White Alcoholic	2.73	0.84	162
	Hispanic Alcoholic	2.57	0.89	159
Make Friends	White No-problem	1.81	0.62	156
	Hispanic No-problem	1.76	0.68	150
	White Alcoholic	2.52	0.83	155
	Hispanic Alcoholic	2.26	0.73	159
Neighbor	White No-problem	1.76	0.65	155
	Hispanic No-problem	1.68	0.69	153
	White Alcoholic	2.36	0.82	155
	Hispanic Alcoholic	2.40	0.82	156
Marry	White No-problem	2.18	0.86	151
	Hispanic No-problem	2.36	1.03	147
	White Alcoholic	3.16	0.81	156
	Hispanic Alcoholic	3.05	0.88	154
Work closely	White No-problem	2.07	0.77	154
	Hispanic No-problem	2.02	0.83	151
	White Alcoholic	3.27	0.76	160
	Hispanic Alcoholic	2.98	0.82	157

Measures are taken on a 4-point scale in response to the question of how willing the respondent is to be close to the vignette's protagonist (1 = “itely willing”:nd 4 = “finitely unwilling)”.

Having seen the black sheep effect for the violation of generic norms quite clearly, the question remains whether it extends to issues consistent with stereotypes of racial outgroups. As shown in Table 3, we find that the black sheep effect does not extend to stereotype-consistent issues and that Hispanic protagonists are consistently evaluated more harshly with respect to violent tendencies and financial mismanagement. White alcoholics are expected to manage their money better than Hispanics 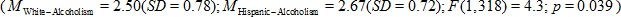. Furthermore, White alcoholics are seen as less violent toward others compared to their Hispanic counterparts 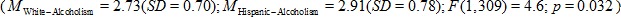.

**Table 3:** Summary Statistics for Stereotype-Consistent Norms.

		Mean	Std. Deviation	*N*
Money	White No-problem	1.49	0.66	157
Management.	Hispanic No-problem	1.39	0.62	153
	White Alcoholic	2.50	0.78	162
	Hispanic Alcoholic	2.67	0.72	157
Violence to	White No-problem	1.85	0.68	158
Others.	Hispanic No-problem	1.85	0.74	151
	White Alcoholic	2.73	0.70	157
	Hispanic Alcoholic	2.91	0.78	153

Stereotypical assessments were measured by the perception of dangerousness toward others (1 = “not at all likely”and 4 = “ery likely” and money management ability (1 = “very able”and 4 = “not able at all”.

We can also consider whether these reactions were triggered by the outgroup tag “Juan” alone or whether it is the tag “Juan” combined with a certain action that activates group bias. Comparing the no-problem with the alcoholic condition shows that the tag “Juan” is not a sufficient condition to trigger negative responses, but that excessive drinking activates group stereotypes. This holds true for perceptions related to money management 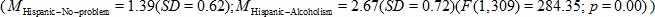 and violence towards others 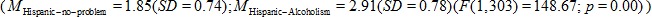.

Lastly, Petersen [25] demonstrated that perceptions of intentionality can influence opinions of outgroups. We check for the possibility that outgroup cues and blame perceptions exert an impact on stereotype-consistent attitudes. The stereotype-consistent attitudes we examine are tendencies towards violence and financial mismanagement. As discussed above, laziness and violence are centerpieces of racial stereotypes [16]. What is more, minorities are disproportionately poor and poverty is a strong predictor of violence, so much so that the effects of poverty and ethnicity on violence are difficult to disentangle statistically [26].

Because emotions and cognitions occur simultaneously, thus making them hard to disentangle [25], we use interaction terms. An interaction between blame and the Hispanic frame is useful here because it elucidates the extent to which the two co-occur when considering problem conditions that are consistent with stereotypes. For example, participants who assign a high level of personal blame for violent behavior may do so all the more when the Hispanic cue is activated and/or a participant who thinks Hispanics are more violent may be more likely to assign blame to them. In other words, blame and the ethnic cue may “operate in a recursive loop,” as suggested generally with respect to emotions and cognitions by Huddy, Feldman, and Cassese [27]. The results presented in Table 4, which examines the alcoholism condition across ethnicities, suggest that blame attribution and outgroup cues exert distinct influences on the assessment of how likely it is that the protagonist is violent towards others. For money management, the racial prime continues to be statistically significant, but blame attribution and the interaction term do not reach statistical significance.

**Table 4:** The Effects of Blame and Race on Stereotype-Consistent Attitudes.

	Violence	Money
Constant	**3.74 (0.39)**	**1.75 (0.43)**
Sex	0.08 (0.09)	**0.192 (0.094)**
Age	0.001 (0.003)	**0.007 (0.003)**
Education	**0.034 (0.016)**	−0.012 (0.018)
Income	−**0.059 (0.025)**	−0.028 (0.028)
Blame	**0.182 (0.063)**	0.027 (0.070)
Hispanic Frame	**0.314 (0.086)**	*0.171 (0.095)*
Blame × Hispanic Frame	−0.118 (0.088)	0.018 (0.098)
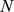	262	273

Data are from the 1996/2006 GSS. All coefficients are un-standardized. Those OLS coefficients and standard errors in bold are statistically significant at or beyond the traditional 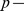
value threshold of 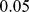 and those that are italicized are statistically significant only at the other common threshold of 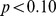. The blame measure has been centered around its mean. Controls include the respondents', sex (coded 1 for female, 0 for male), age (in years), education (coded 1 for at least a high school degree, and 0 otherwise), and income (12 income categories).

Some have suggested that gender, rather than arbitrary group membership (race in this case), is the primary basis of evaluations of individual behavior [28]. To examine this possibility, we also analyzed the vignette protagonist's gender. Indeed, White male alcoholics are blamed more 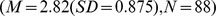 than their Hispanic counterparts 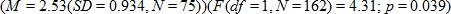. In contrast, females receive less blame regardless of their race 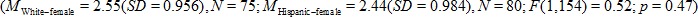. Taken together these results suggest that if a white male deviates from a group norm, he is judged more harshly than if a Hispanic or a woman would be. However, the gender related differences could not be replicated for the stereotype-consistent measures. Also, splitting the respondents by gender does not lead to different results (not reported).

## Discussion

The results largely support the black sheep effect suggested by SIT. Our strongest evidence for this finding is that White alcoholics are blamed for their condition more than Hispanic alcoholics. Beyond that, analyses of many of the social distance measures – all of those implying a direct relationship with the vignette's protagonist – corroborate this result, with insignificant results wherever there are not supportive results. However, the black sheep effect does not seem to extend to the realm of issues consistent with outgroup stereotypes. Hispanic protagonists were seen to be more prone to violence against others and an inability to manage their money. Although it has been reported that negative outgroup attitudes, specifically with respect to immigrants, can be dampened when considering an individual as opposed to an entire group [24], [29], we found that stereotype-consistent attitudes were applied to individuals as well. Though one may object that the racialized cue word “Juan” might have triggered this result, the direct comparison to the Hispanic no-problem condition showed that such a cue word is not sufficient to trigger the reaction. The implication is that actions matter and only in the presence of the action and the cue are more negative assessments of Hispanics elicited with respect to violent tendencies and money management. This interpretation of the results is consistent with the black sheep effect but extends its reach: we show that negative assessments of the outgroup are also applied at the individual level.

Although the GSS Module did not include emotion measures, and so we cannot explicitly test all elements of intergroup emotion theory, the results provide some insights into studies of emotions and appraisal. Contrary to appraisal-based intergroup emotion theories that predict qualitatively distinct emotions directed towards different groups (e.g. [17]), we showed that outgroup cues are, at best, context dependent and do not seem to elicit unique emotions that influence evaluations. If the predictions of IET had been manifest, we would have observed the outgroup receiving consistently more negative responses across all items. One counter argument may be that positive emotions, such as pity, could be elicited in this process. While it seems blame should offset pity (e.g. one feels less pity the more blame one assigns). Of course, the apparent inconsistency with IET does not diminish the importance of emotions; we suspect that emotional measures more accurate than self-reports [30] would provide considerable insight into the dynamics of individual evaluations.

Our results suggest that the null predictions that follow from priming studies (e.g. [10]) need to be adapted based on the different evaluation criteria available to the respondents. Race and blame seem to constitute distinct evaluation criteria that subjects apply independently to different issues. Cognitive theories need to account for the dynamics of ingroup and outgroup cues, as well as individual blame attributions, as variable evaluation criteria. While research based on evolutionary psychology has found that intention strongly influences attitudes on criminal justice issues [25], this study supports, but also extends, those findings. The results show that blame attributions are associated with violence assessments as we would expect, but that this association does not extend to money mismanagement. Race, however, seems to affect evaluations of both violent propensities and money management, but only if a contextual opportunity presents itself (norm violation). That individuals use different evaluation criteria depending on the issue at hand highlights the need for a more dynamic approach because blame and race operate on the same issue with seemingly different processes. This stands as a challenge to future research.

## Conclusions

While a valuable body of research has studied the extent to which issues are racialized, typically welfare and crime, fewer studies have addressed the question of how non-racialized issues are affected by racial cues. This study took the non-racialized norm violation of alcohol abuse and asked whether group cues and/or individual action affect individuals' attitudes. The results show that action matters more in influencing attitudes than group cues, but that group bias interacts with those assessments. Group cues alone do not exert any impact. This supports the contextual importance and cognitive basis of individuals' evaluations.

The published work in political psychology has mostly focused on the group level and has demonstrated the negative impact of outgroup cues on group evaluations or group-related measures, but the possibility that norm violations by members of the ingroup might be judged more harshly has been largely neglected. When considering only the modal category of group-based studies, we risk the ecological fallacy that the same processes affect individuals of different races. The results of this study highlight the need for a more dynamic approach to the study of racial cues and cognitive attribution in political psychology.

Individuals' evaluations seem to occur dynamically by switching from one evaluation criteria to another. Blame and race are sometimes used as distinct evaluation criteria based on the situation being evaluated. Future studies should account for the possibility that group bias is issue dependent, thus enhancing our understanding of how far this dynamic process reaches. One possibility would be to use Implicit Association Tests (IAT) on various issues [31] in order to shed light on the dynamics of the issue categories individuals use to make their evaluations.

Future studies may extend this research on other dimensions as well. While we examined individual norm violation, the impact of racial cues at the group level might be tempered [29]. This is an important possibility because individuals usually evaluate groups and not individuals when forming attitudes on public policies [24]. However, studies often find that individual level exposures, such as actual language exposure [9] or racial proximity [32], can impact attitudes on group policies. Furthermore, IAT tests might solve the puzzle as to whether “race” and/or “blame” are used as cues to evaluate policies in this context.

A final avenue of potential research concerns sampling. While the Hispanic sample in the GSS was too small to be included in this study, future studies could extend their sample to Hispanics and other racial groups in order to explore whether the same black sheep dynamics hold in minority groups as well as majority groups.

## Supporting Information

Supporting Information S1 **Exact question wording.**
(PDF)
