# Supplementary material for: The Impact of Hispanic and White Group Cues on Attitudes Towards the Violation of Generic Norms
Source: PLoS One. 2013 Dec 27;8(12):e83154. doi: 10.1371/journal.pone.0083154 (PMC3873910; doi:10.1371/journal.pone.0083154)
Supplement: Supporting Information S1 — Exact question wording. (PDF) [file pone.0083154.s001.pdf]

Supporting Information: The Impact of Hispanic and White Group Cues on Attitudes Towards the Violation of Generic Norms

Mirjam Cranmer[1], Skyler J. Cranmer[2,3]
1 Social Science Research Institute, Duke University, Durham, North Carolina, USA
2 Department of Political Science, University of North Carolina at Chapel Hill, Chapel Hill, North Carolina, USA
3 Alexander von Humboldt Fellow, University of Konstanz, Germany
∗ E-mail: skyler@unc.edu

S1. Question Wording

Wording for the no-problem condition: [John (condition 1) / Juan (condition 3)] is a [White/Hispanic] man with an [eighth grade/high school/college education]. Up until a year ago, life was pretty okay for [John/Juan]. While nothing much was going wrong in [John's/Juan's] life he sometimes feels worried, a little sad, or has trouble sleeping at night. [John/Juan] feels that at times things bother him more than they bother other people and that when things go wrong, he sometimes gets nervous or annoyed. Otherwise [John/Juan] is getting along pretty well. He/ enjoys being with other people and although [John/Juan] sometimes argues with his family, [John/Juan] has been getting along pretty well with his family.

Wording for the alcoholic condition: [John (condition 2) / Juan (condition 4)] is a [White/Hispanic] man, who has completed [the 8th grade/high school/college]. During the last month [John/Juan] has started to drink more than his usual amount of alcohol. In fact, he has noticed that he needs to drink twice as much as he used to to get the same effect. Several times, he has tried to cut down, or stop drinking, but he can't. Each time he has tried to cut down, he became very agitated, sweaty and he couldn't sleep, so he took another drink. His family has complained that he is often hung-over, and has become unreliable making plans one day, and canceling them the next.
